# Evaluation of Usefulness of AlCrN Coatings for Increased Life of Tools Used in Friction Stir Welding (FSW) of Sheet Aluminum Alloy

**DOI:** 10.3390/ma13184124

**Published:** 2020-09-16

**Authors:** Piotr Lacki, Wojciech Więckowski, Grzegorz Luty, Paweł Wieczorek, Maciej Motyka

**Affiliations:** 1 Department of Civil Engineering, Faculty of Civil Engineering, Czestochowa University of Technology, ul. J.H. Dabrowskiego 69, 42-201 Czestochowa, Poland; 2 Department of Technology and Automation, Faculty of Mechanical Engineering and Computer Science, Czestochowa University of Technology, ul. J.H. Dabrowskiego 69, 42-201 Czestochowa, Poland; wieckowski@itm.pcz.pl; 3 Technical Department, Developement Projects Office, European and Space Projects Section, PZL Mielec/A Lockheed Martin Company, ul. Wojska Polskiego 3, 39-300 Mielec, Poland; grzegorz.luty@lmco.com; 4 Department of Materials Engineering, Faculty of Production Engineering and Materials Technology, Czestochowa University of Technology, ul. J.H. Dabrowskiego 69, 42-201 Czestochowa, Poland; pawel@wip.pcz.pl; 5 Department of Materials Science, Faculty of Mechanical Engineering and Aeronautics, Rzeszow University of Technology, Al. Powstancow Warszawy 12, 35-959 Rzeszow, Poland; motyka@prz.edu.pl

**Keywords:** tool wear, PVD coating, AlCrN coating, friction stir welding (FSW), Al 7075-T6 alloy

## Abstract

The study presents the results of examinations of wear in tools made of 1.2344 steel without and with an anti-wear coating in the process of welding overlap joints of sheet metal made of 7075-T6 aluminum alloy using friction stir welding (FSW) technology. A commercial anti-wear AlCrN coating (Balinit^®^ Alcrona Pro by Oerlikon Balzers Coating Poland Sp. z o.o., Polkowice, Poland) was examined, applied using physical vapor deposition (PVD) and used to improve tool life in metalworking processes. Wear tests for the tools were conducted in industrial conditions at specific parameters of the friction stir welding process. Tool wear was evaluated through examination of the tool working surface. The results of the static tensile strength tests and metallographic examinations of the joints were used to evaluate the effect of tool wear and the coating impact on joint quality. The results obtained in the study show that the tool made of 1.2344 steel was intensively worn after the welding of a joint with the length of 200 m, increasing the risk associated with further use of the tool and suggesting the tool’s low durability. The use of the AlCrN coating led to an increase in tool life. The coating limits the process of tool wear and can be used as an anti-wear coating for tools used in the FSW of aluminum alloys.

## 1. Introduction

Friction stir welding (FSW) technology allows for welding joints with good mechanical and structural properties, and therefore it has been widely used in many industries, e.g., automotive, defense, energy, aerospace, rail and machine building sectors [1,2,3]. A significant problem hindering the joining of materials in FSW is the low durability of the tools [4]. During such processes, the tool is exposed to high mechanical and thermal loads and is subjected to wear as a result of abrasion, plastic deformation, adhesion and chipping.

The intensity of tool wear depends on the type of tool material in combination with the welded material, tool design and welding parameters [3,5,6]. Tool wear leads to modification of the geometry of the working part of the tool, which, along with properly chosen process parameters, is responsible for joint quality [7,8,9,10]. The reasons for defect formation in FSW joints have already been discussed [11], as has the possibility of defect detection using ultrasonic testing [12].

As reported in [13,14], waste products of tool wear, which are transferred to the weld, lead to defects and deterioration of joint quality. Therefore, tool life, expressed by the time of its operation or the weld length, is strongly dependent on these factors and corresponds to a given set of welding process parameters.

The improvement of the functional properties of tools can be achieved through the development of new grades of materials, the use of appropriate chemical compositions and the application of modern methods and technologies for their production using thin protective (anti-adhesive, anti-wear) coatings, including modern multilayer coatings and termed multilayers [15,16,17]. Due to the variety of working conditions, tool designs and dominant types of wear, technological coatings used to enhance tribological properties have to be deposited using specific manufacturing methods.

Anti-wear coatings have been successfully used for cutting tools, with their main purpose being to reduce tool wear in the contact area between the tool and material being processed. They also improve resistance to the chemical effect on the surface layers of the tool, form a barrier to prevent heat penetration into the tool, reduce diffusion, increase the hardness of surface layers on working surfaces and reduce the coefficient of friction [18,19,20,21]. In contrast to machining, features such as the increased coefficient of friction and having no limitation in the temperature rise in the welding zone improve the FSW process quality. Therefore, the choice of coatings for FSW tools represents a major challenge.

Coatings often exhibit significantly different properties. Therefore, it is necessary to perform experimental tests to evaluate the mechanical and tribological properties to choose the optimal coating for a specific application.

Proper choice of a coating requires an analysis of the conditions in which the coating will be used and should be preceded by a series of experimental studies [22,23,24,25]. When choosing the type of coating for FSW tools, it is necessary to follow the requirements for coatings dedicated for working in conditions of dry friction at elevated temperatures. A properly chosen coating allows for manufacturing tools from cheaper materials with lower functional properties, while obtaining better parameters of tool surfaces and increasing their life.

Literature reports provide information about the use of anti-wear coatings in FSW tools. However, no details have been published on the durability of such coatings and, consequently, on the improvement of tool life and extending their period of use.

The results of examinations of an AlCrN anti-wear coating on the working surface of an FSW tool made of tungsten carbide were presented in the study by Casalino et al. [25]. The coating used by these researchers allowed for obtaining joints without defects; however, the author failed to provide information about its effect on tool life. In the study [23], Batalha et al. presented the results of examinations of wear on an AlCrN coating deposited on the surface of an FSW tool made of cemented carbides in the process of titanium welding. The tests revealed total wear of the coating used. The coating did not meet expectations in terms of improving tool durability. The results of tests confirming improvements in the durability of steel tools used in the FSW of metal composite materials due to the use of the anti-wear TiAlN coating were presented by Devanatha et al. [26]. Adesina et al. presented the results of examinations of wear of a steel tool with an AlCrN coating used in the process of FSW of 6061-T6 aluminum alloy. The researchers demonstrated the usefulness of the coating, which both limited tool wear and helped obtain the joint without defects [24]. Information about the use of the anti-wear coating made of boron carbide B4C in tools made of tool steel for the friction stir welding of metal matrix composites (MMCs) was presented by Bhat et al. [27]. The authors did not present, however, information about potential improvements in tool life due to the use of the coating.

The present study aimed to evaluate the usefulness of an anti-wear coating in terms of the application and the improvement of tool life during the FSW of 7075-T6 aluminum alloy sheet metals. Based on the analysis of tool operating conditions and technical specifications of coatings, a commercial AlCrN coating (Balinit^®^ Alcrona Pro) produced by physical vapor deposition (PVD) was selected for testing. The coating was used to improve the durability of tools used in the processing of metals operating at high mechanical and thermal loads.

## 2. Materials and Methods

The preliminary tests were aimed at assessing the tribological properties and resistance to tribological wear of the selected technical coating deposited on the base material of the sample. Commercially available AlCrN, TiN, TiAlN and TiCN coatings commonly used for metalworking tools were tested. The choice of the coating to be applied on the tool working surface was based on the results of friction and wear tests [28]. The friction and wear tests of the selected coating were performed using T-05 block-on-ring and T-01 pin-on-disc testers.

Samples for testing using the T-05 tester were 6 × 15 × 10 mm^3^ blocks and were coupled with a counter-specimen in the form of a ring with a diameter of 35 mm and a width of 9 mm, made of bearing steel. The samples for testing using the T-01 tester were discs with a diameter of 39 mm and a thickness of 6.5 mm and were coupled with a counter-specimen in the form of a 3 mm diameter pin made of tungsten carbide WC.

The coating was deposited onto the working surfaces of the samples in the form of discs and blocks made of hot work tool steel (1.2344). Before the coating was applied, the samples were heat-treated to ensure a hardness of approximately 50 HRC. The samples and tools made of 1.2344 hot work tool steel were quenched from the temperature of 1100 °C and double tempered at 580 °C with air cooling. After the coating was applied, microhardness measurements were taken on the surface of the samples, which was ~1300 HV0.1 (~70 HRC).

The tests included recording the friction force to determine the coefficient of friction. Friction and wear tests on the T-05 block-on-ring tester were carried out on the friction distance of 50 m with a concentrated contact, at a sliding speed of 0.1 m/s and a load of 50 N. Friction and wear tests on the T-01 pin-on-disk tester were carried out under technically dry friction conditions at a sliding speed of 0.1 m/s and a pin load of 20 N.

The surface wear of the tested samples was assessed by measuring the wear trace profile with the Taylor Hobson Polska TALYSURF 120 profilometer (Warsaw, Poland). The adhesiveness of the coating was evaluated by means of the scratch test. The tests were carried out using the Revetest Xpress device (CSM Instruments, Peseux, Switzerland) at a steady rate of increase in the load of the indenter (from 1 to 100 N) over a distance of 10 mm and a sample speed of 6.06 mm/min.

**Figure 1:**
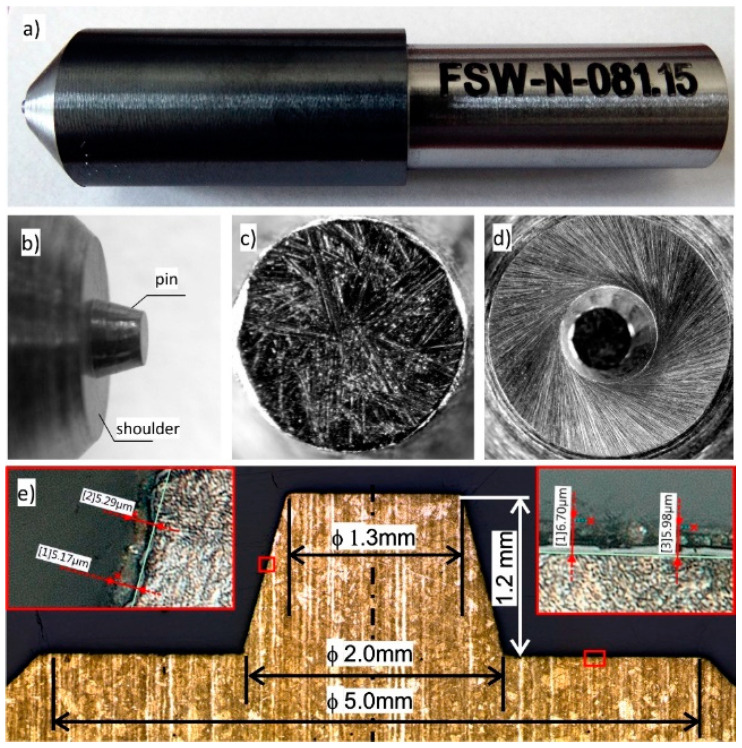
Friction stir welding (FSW) tool with the AlCrN coating used for wear tests: (**a**) tool, (**b**) tool working parts, (**c**) frontal surface of a pin, (**d**) shoulder surface, (**e**) cross-section of the tool.

The main research included wear tests of FSW tools without a coating and with an applied protective coating. The examinations were conducted in industrial conditions. The FSW tools, with geometry developed at the Czestochowa University of Technology, were made of 1.2344 hot work tool steel at the Medical Tool Factory CHIRMED in Rudniki, Poland. The tool made of 1.2344 steel with the deposited AlCrN coating is shown in Figure 1.

The measurements of the thickness of the obtained coatings were made on a cross-section using secant lines. Coatings were deposited on both the working surface of the samples and the tool working surfaces in Oerlikon Balzers Coating Poland Sp. z o.o. (Polkowice, Poland).

During the wear tests, overlap joints were made of 7075-T6 aluminum alloy sheets with a thickness of 1.0 mm for the top sheet of metal and 0.8 mm for the bottom sheet of metal. The parameters of the welding process were as follows: rotational speed of the tool *n* of 1000 rpm, welding velocity *v* of 200 mm/min and tool depth of 1.2 mm. Joints were welded using a DMC 104V machine in PZL Mielec/A Lockheed Martin Company (Mielec, Poland).

**Figure 2:**
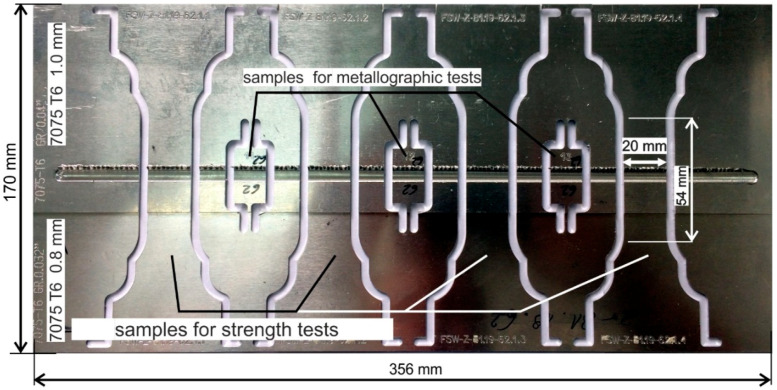
Joint welded during wear tests of FSW tools.

Two research stages were planned for each of the tested tools. After each stage, when joints with a total length of approximately 100 m were welded, the tool was chemically cleaned (to remove welded material), and a visual inspection of the state of the working surface was performed. After the next approximately 20 m of welding, the samples were cut from the joints for strength and metallographic tests in order to assess the effect of the protective coating and tool wear on the weld quality (Figure 2).

In order to confirm the presence of the deposited protective coating on the tool surface, the chemical composition of the tool’s working surface was analyzed after each research stage using the Jeol JSM-6610LV scanning electron microscope (Akishima, Japan) with the LaB6 filament and an EDS attachment for chemical analysis (SEM/EDS).

The evaluation of wear of the tested FSW tools was based on the comparison of the contour of a new tool with the contour of the tool subjected to wear tests after successive research cycles. The geometry of the tool working part was measured using the OGP Smart Scope FLASH 200 multisensor measurement system (Rochester NY, USA). Absolute changes of selected geometrical parameters of the tool working part were evaluated in relation to the joint length (in subsequent stages of research), i.e., changes in the diameter and height of the pin [29].

After the completion of the next stage of the tool work, a visual assessment of the tool working surface was made based on the photographs obtained from the stereoscopic examination. The effects of tool wear observed on the joint included changes in the weld quality (e.g., the presence of defects or variable load capacity of the joint).

## 3. Results and Discussion

**Figure 3:**
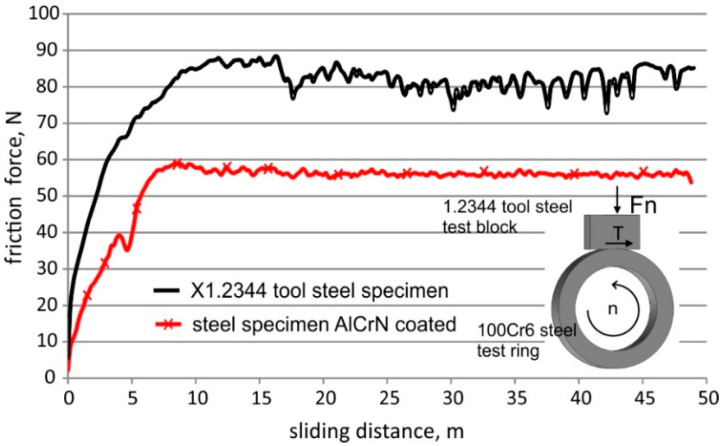
Diagram of changes in friction force vs. friction distance (T-05 tester).

**Figure 4:**
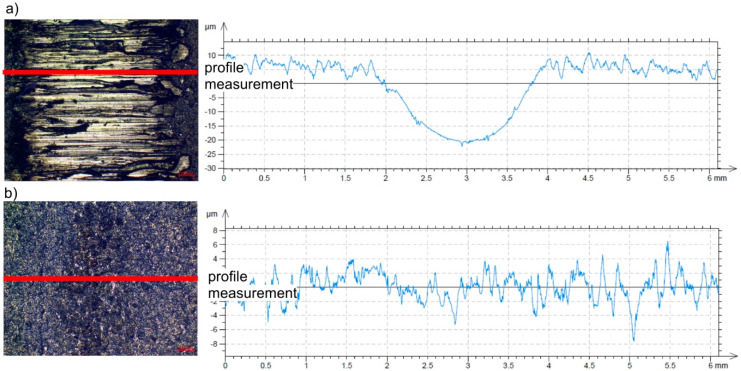
View and profile of the friction marks formed on the surface of the samples during friction and wear tests (T-05 tester): (**a**) 1.2344 tool steel specimen, (**b**) AlCrN-coated steel specimen.

The results of the experimental tests confirmed that the application of the AlCrN coating significantly reduced the consumption of the base material. The recorded patterns of changes in the friction force vs. friction distance and the images of the friction marks on the steel surface of the specimens with and without the AlCrN protective coating recorded on the T-05 tester are shown in Figure 3 and Figure 4, respectively.

The values of the coefficient of friction were determined on the basis of the friction force measurements recorded during the tests using the T-01 tester. The application of the anti-wear AlCrN coating led to a reduction in the coefficient of friction compared to that observed during testing of the sample without the coating (µ = 0.40–0.45). Its value increased from 0.25 to 0.40 as the coating wore off. The changes in the coefficient of friction with the increasing degree of wear of coatings may lead to changes in the conditions of the FSW process during tool operation and are not beneficial due to the course of the process.

The value of critical load LC2 that characterizes adhesion to the base material of the AlCrN coating evaluated using the scratch test method was 81 N.

**Figure 5:**
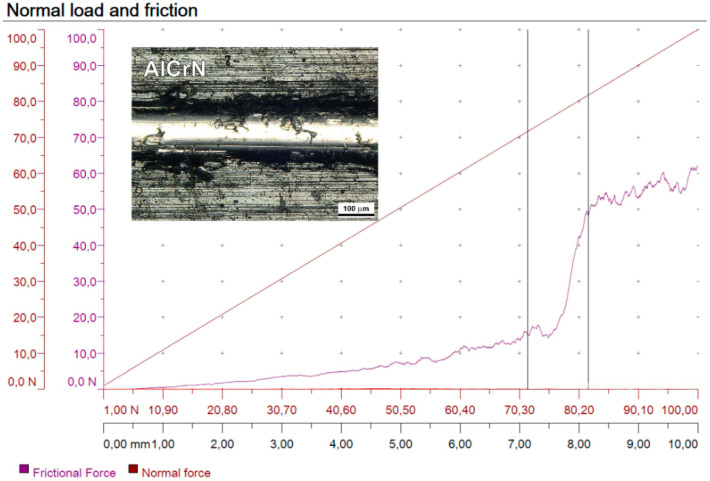
Scratch test results of the X40CrMoV5-1 tool steel sample with AlCrN coating.

The profiles of normal force F_N_, friction force F_T_ and the coefficient of friction µ are presented in Figure 5.

**Figure 6:**
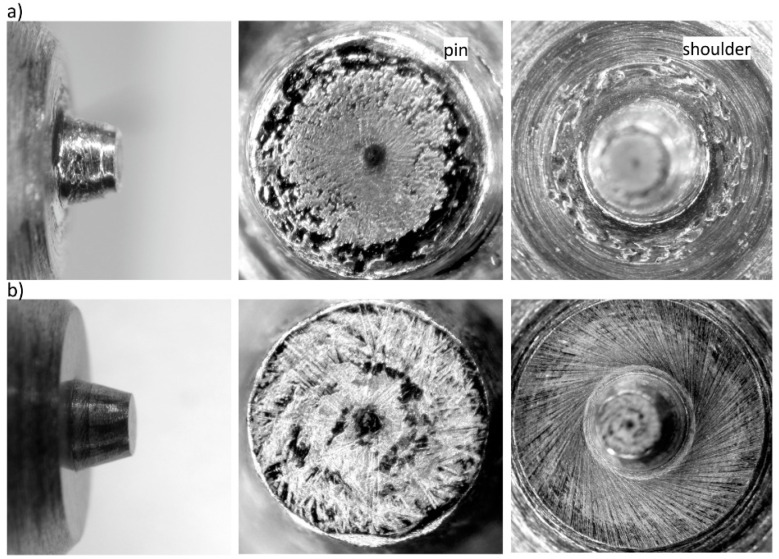
Tool working surfaces (pin and shoulder) after the first stage of testing (joint length of approximately 100 m): (**a**) tool made of 1.2344 steel, (**b**) tool made of AlCrN coated 1.2344 steel.

The tool working surfaces after the first stage of testing are presented in Figure 6.

In contrast to the tool without a protective coating, the inspection of the tool with the AlCrN coating revealed no marks suggesting tribological wear or areas indicative of degradation of the coating on the tool surface.

**Figure 7:**
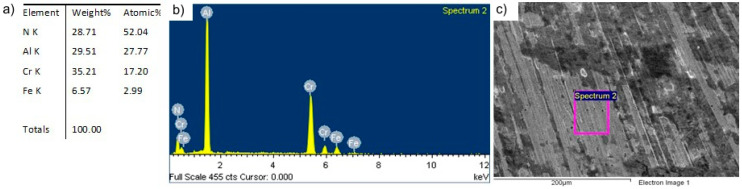
SEM/EDS analysis of the chemical composition of the shoulder surface of the tool with the AlCrN coating after the first stage of testing: (**a**) content of elements, (**b**) EDS spectrum, (**c**) surface analyzed.

Analysis of the chemical composition of the tool surface after its use confirmed the presence of a coating on the tool surface, as evidenced by the high mass ratio of the elements that form the coating, i.e., N, Al and Cr. The presence of iron is from the layer and smaller part of the tool. The results of the chemical analysis of the surface layer of the tool’s working surface are shown in Figure 7.

**Figure 8:**
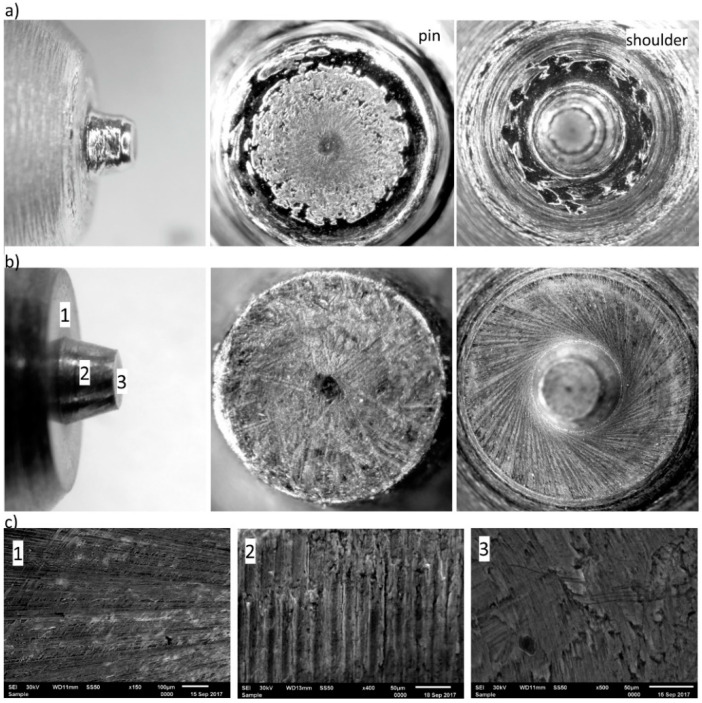
Tool working surfaces (pin and shoulder) after the second stage of testing (joint length of approximately 200 m): (**a**) tool made of 1.2344 steel, (**b**) tool made of AlCrN-coated 1.2344 steel, (**c**) microscopic images (SEM) of the tool surface after machining. (**1**) shoulder surface, (**2**) side surface of a pin, (**3**) frontal surface of a pin.

The tool working surfaces after the second stage of testing are presented in Figure 8.

Significant material loss can be observed on the working surface of the tool without a coating, with numerous depressions, grooves and circumferential scratches. Examination of the tool surface with the AlCrN coating showed no signs of wear or damage to the coating.

**Figure 9:**
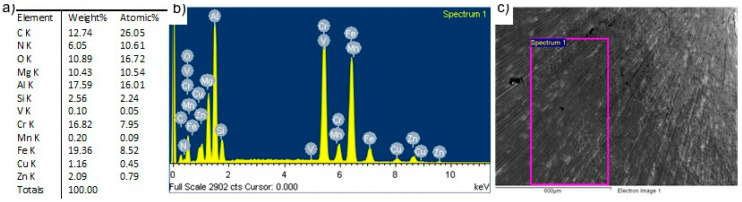
SEM/EDS analysis of chemical composition of the shoulder surface of the tool with the AlCrN coating after the second stage of testing: (**a**) content of elements, (**b**) EDS spectrum, (**c**) surface analyzed.

The SEM/EDS analysis of the chemical composition of the working surface of the tool showed the presence of the AlCrN protective coating (Figure 9).

The EDS analysis also evaluated the volume of the base material (presence of Fe), which may suggest a gradual wear of the coating. The loss of tightness of the coating resulted in sealing the cracks with the welded material, hence the presence of such elements as Cu, Al, Si, Zn and Mg.

**Figure 10:**
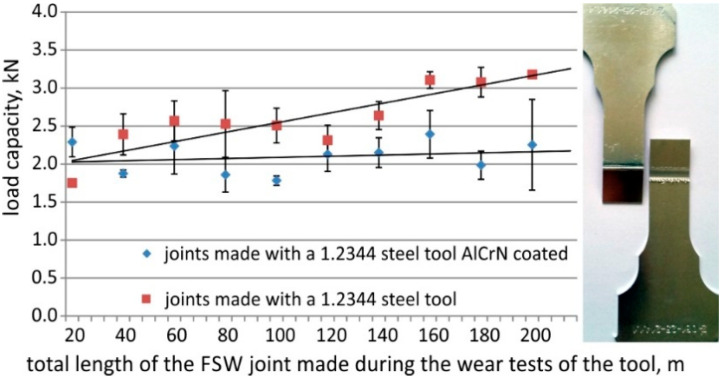
Strength of FSW test joints welded during wear tests of steel tools without a coating and with the AlCrN protective coating.

The results of strength tests of the FSW joints welded during the wear tests are presented in Figure 10.

FSW joints welded with new tools had similar strength, at the level of 2 kN. In the case of FSW joints welded using a tool without a coating, differences and a large spread of tensile strength values resulting from intensive tool wear were observed. The increase in tool wear led to an increase in joint strength and a decrease in its spread. Changing the shape of the tool caused modification of the geometric parameters of the weld (increase in the weld width measured at the level of the contact line of the joined plates), which was important from the standpoint of their strength.

**Figure 11:**
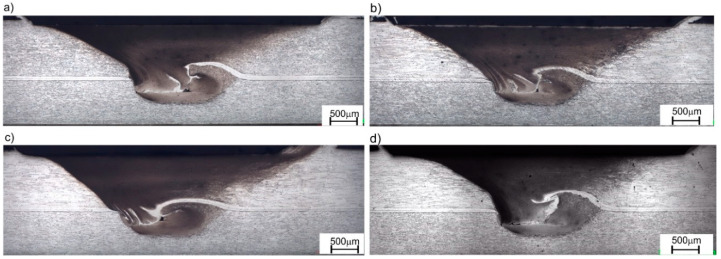
Cross-section of FSW joints welded: (**a**) FSW joint made with a new tool made of 1.2344 steel, (**b**) FSW joint made with a new AlCrN-coated tool (1.2344), (**c**) FSW joint made with a worn-out tool (1.2344)—total length of FSW joint: 200 m, (**d**) FSW joint made with an AlCrN-coated worn-out tool (1.2344)—total length of FSW joint: 200 m.

In the case of FSW joints welded using the tools with the protective coating, their strength ranged from 1.8 to 2.4 kN over the entire duration of the wear tests. No significant changes in the geometric parameters of the joints were observed in this case. It is important to note that the strength of the tested joints was also affected by defects identified in the cross-section of the joints during metallographic examinations (Figure 11).

## 4. Conclusions

The study demonstrates that the quality of joints is affected by both the degree of tool wear (changing the geometry of the working surface) and the presence of a protective coating (changing friction conditions), responsible for the transport of the plasticized material and the amount of heat generated in the weld.

The wear of FSW tools made of 1.2344 steel without protective coating was manifested by a change in the geometry of the working part due to mechanical wear and adhesion. The study indicated that the maximum tool wear occurred on the lateral surface of the tool shank (uneven radial wear of the pin), and insignificant wear occurred on the shoulder surface and the pin, measured in the direction of the tool axis. The change in the geometry of the working part of the tool due to its wear allowed for obtaining joints with higher strength than those obtained with the new tool at the initial stage of testing.

Intensive wear of the tool working surface without a protective coating led to changes in the geometrical parameters of the joint, such as weld nugget width, stirring depth of the bottom sheet material and material thinning in the top sheet metal. As the uncoated tool wear progressed, the joints obtained during the test were characterized by an increased width of weld nugget from 1.4 to 1.6 mm and an increased stirring depth in the material of the bottom sheet metal from 0.4 to 0.5 mm, which resulted in an increase in the tensile strength of the joints obtained during the final stage of the test compared to the samples taken from the joints obtained using the new tool. Both the weld width and the mean material stirring depth in the area of lower sheet metal in the joints made with the AlCrN-coated tool remained constant at ~1.4 and ~0.5 mm, respectively.

The effects of the accelerated wear of the uncoated tool included variation in the tensile strength values for FSW joints, ranging from 1.7 kN for joints made with the new tool to 3.2 kN for joints made in the final stage of testing, and a large spread of the tensile strength values (about 14%) in the control joints group.

Both the experimental tests and the FSW tool tests conducted in industrial settings demonstrate that an increase in the durability of tools made of 1.2344 steel can be achieved by properly selected coatings that reduce wear.

The presence of the AlCrN coating on the tool working surface after the test stage shows that the applied protective coating ensures the unchangeability of the tool’s geometry during operation, thus improving the tool life.

Maintaining constant dimensions of the working part of the tool ensures constant conditions of the FSW process and allows for obtaining joints with reproducible strength parameters.

Experimental examinations showed that the use of the AlCrN coating led to a reduction in the coefficient of friction, which has a significant effect on the FSW process. The research in industrial settings demonstrated that the joints obtained using the tool with anti-wear coatings have lower strength compared to the joints obtained with uncoated tools, with the other FSW process parameters remaining steady.

The joints made during the wear tests of the tool with the AlCrN coating showed an average strength at a relatively low level of ~2.1 kN. Both the weld width and the mean material stirring depth in the area of lower sheet metal in the joints made with the AlCrN-coated tool remained at a level of ~1.4 and ~0.5 mm, respectively.

The strength of the joints welded during the wear tests of tools without coatings and with the AlCrN coating is also due to the presence of defects in the weld cross-section found during metallographic tests. Therefore, in order to further improve the quality of the joints (eliminate defects and increase their strength), further research is needed to optimize the shape of the working part of the tool and to select the remaining parameters of the FSW process after applying appropriate anti-wear coatings.
